# Clinical utility of aVR lead T-wave in electrocardiogram of patients with ST-elevation myocardial infarction

**DOI:** 10.1186/s12872-021-02335-5

**Published:** 2021-10-28

**Authors:** Babak Kazemi, Seyyed-Reza Sadat-Ebrahimi, Abdolmohammad Ranjbar, Fariborz Akbarzadeh, M. Reza Sadaie, Naser Safaei, Mehdi Esmaeil zadeh-Saboor, Bahram Sohrabi, Samad Ghaffari

**Affiliations:** 1 Cardiovascular Research Center, Madani Hospital, Tabriz University of Medical Sciences, Tabriz, Iran; 2 NovoMed Consulting, Germantown, MD 20874 USA

**Keywords:** ST-elevation myocardial infarction, Electrocardiogram, T wave, aVR lead, Prognosis

## Abstract

**Background:**

aVR lead is often neglected in routine clinical practice largely because of its undefined clinical utility specifications. Nevertheless, positive T-wave in aVR lead has been reported to be associated with poor clinical outcomes in some cardiovascular diseases. This study aimed to prospectively investigate the prognostic value and clinical utility of T-wave amplitude in aVR lead in patients with acute ST-elevation myocardial infarction (STEMI).

**Methods:**

A total of 340 STEMI patients admitted to a tertiary heart center were consecutively included. Patients were categorized into four strata, based on T wave amplitude in aVR lead in their admission ECG (i.e. < − 2, − 1 to − 2, − 1 to 0, and ≥ 0 mV). Patients’ clinical outcomes were also recorded and statistically analyzed.

**Results:**

In-hospital mortality, re-hospitalization, and six-month-mortality significantly varied among four T wave strata and were higher in patients with a T wave amplitude of ≥ 0 mV (*p* 0.001–0.002). The groups of patients with higher T wave amplitude in aVR, had progressively increased relative risk (RR) of in-hospital mortality (RRs ≤ 0.01, 0.07, 1.00, 2.30 in four T wave strata, respectively). T wave amplitude in the cutoff point of − 1 mV exhibited a sensitivity and specificity of 95.83 (95% CI 78.88–99.89) and 49.68 (95% CI 44.04–55.33).

**Conclusion:**

Our study demonstrated a significant association of positive T wave in aVR lead and adverse clinical outcomes in STEMI patients. Nevertheless, the clinical utility of T-wave amplitude at aVR lead is limited by its low discriminative potential toward prognosis of STEMI.

**Supplementary Information:**

The online version contains supplementary material available at 10.1186/s12872-021-02335-5.

## Introduction

ST-elevation myocardial infarction (STEMI) is characterized as one of the leading causes of mortality and morbidity worldwide. The 30-day mortality rate of STEMI is estimated to be 2.5–10% in developed countries, with the highest rates attributable to patients aged over 75 years [1]. Twelve lead electrocardiogram (ECG) is one of the most commonly used tools in the diagnosis and prognosis of STEMI. A variety of prognostic criteria based on the ECG findings in STEMI patients have been introduced [2–4]. However, aVR lead (augmented unipolar right arm lead) is often neglected in routine clinical practice, largely because of its undefined clinical utility specifications for a reliable prognosis and definite diagnosis of myocardial infarction [5, 6]. Nevertheless, the ECG parameters (such as P, QRS, T waves, and ST-segment) in aVR lead are often characteristics in different conditions, and therefore, correct interpretations of these changes were proposed to increase the accuracy of clinical diagnosis and prognosis [6]. For instance, some studies have reported a significant association of ST-segment changes in aVR lead with worse prognosis in patients with STEMI [7–10]. Moreover, positive T-wave profiles in aVR lead in patients with heart failure [11], old anterior myocardial infarction [12], and even in general population [5] are reportedly associated with poor clinical outcome and considerably high cardiovascular mortality rate in six months [5, 11, 12]. However, the prognostic value of T-wave amplitude changes in patients with acute STEMI remains unclear. Therefore, in this study, we aimed to evaluate the clinical utility of T-wave amplitude in aVR lead toward a better prediction of short and midterm outcomes of patients with acute STEMI.

## Methods

### Study population

In this prospective study, a total of 410 patients were evaluated and datasets from 340 patients were entered in the final analysis (70 patients were excluded based on the exclusion criteria, Additional file 1: Figure S1). All adult patients (aged 18 years or older) with any gender with a definite STEMI diagnosis for the first time who were admitted because of chest pain within 24 h since the initiation of symptoms at a tertiary heart center (the name of the center is undisclosed for peer review) were consecutively included from April 2020 to September 2020. Patients were diagnosed as STEMI if they had typical ischemic chest pain lasting ≥ 20 min and ST-elevation of 0.25 mA or more at the J point in men below the age of 40 years, ≥ 0.2 mV in men over the age of 40 years, or ≥ 0.15 mV in women in leads V2–V3 and/or ≥ 0.1 mV in other leads (in those without left ventricular hypertrophy or left bundle branch block) on the admission ECG, with/without an increase in cardiac enzyme concentrations (troponin I [CTNI] and/or creatine phosphokinase-MB [CKMB]) [13]. Patients with atrial fibrillation or flutter, left ventricular hypertrophy, right or left bundle branch block, implanted pacemaker or defibrillator, or Wolf-Parkinson-White syndrome were excluded due to imposing additional ECG alteration. Informed consent was obtained from all patients, and the study was conducted in accordance with the declaration of Helsinki [14]. The study protocol was approved by the medical ethics committee of our institution (the name of the institution and ethical code is undisclosed for peer review). The demographics, medical history, laboratory test results, ECG, echocardiographic and angiographic findings were documented for all patients. Patients were categorized into four strata, based on T wave amplitude in aVR lead in their first ECG at admission to the emergency room (Additional file 1: Figure S2):

1. Patients with T wave amplitude of less than -2 mV
2. Patients with T wave amplitude of − 1 to − 2 mV
3. Patients with T wave amplitude of − 1 to 0 mV
4. Patients with T wave amplitude of ≥ 0 mV

The follow-up after exiting hospitalization was performed by telephone interviews, regular visits, and hospital records evaluation.

### Study endpoints

The primary endpoint was in-hospital mortality, defined as the occurrence of death due to heart attack/STEMI during the exiting hospital stay. The secondary endpoints were the length of hospital stay (defined as the number of days from admission to discharge/death), the number of patients who develop ventricular tachycardia (VT; sustained and non-sustained) or ventricular fibrillation (VF) during the hospital stay, re-hospitalization (defined as re-admission because of either myocardial infarction or decompensated heart failure until six months from onset of disease), and six months cardiovascular mortality (defined as the occurrence of death due to cardiovascular causes in six months from onset of disease).

### Electrocardiogram (ECG)

All 12-lead ECGs were obtained using MAC 500 ECG machine (GE medical system, USA) with a paper speed of 25 mm/s and standard voltage. ECG analyses were conducted by an attending cardiologist who was blinded to the patients’ clinical status, using digital calipers on a 12-lead ECG and magnified to 200% of normal size. The ECG analysis was repeated by another attending cardiologist and the discrepancies were resolved by consultation with the third cardiologist to mitigate intra-observer variability.

The T-wave amplitude was measured as the value of the largest deflection above and below the baseline in a window spanning from 80 ms after the end of QRS to the end of the T wave. The infarction location was determined based on ECG findings.

### Other tests

In addition to CTNI, creatinine (Cr) measurements, and complete blood tests, routine clinical and paraclinical quantitative measurements were undertaken per standard procedures. Echocardiographic evaluations were conducted at the time of admission to the emergency room. Two-dimensional transthoracic echocardiography was performed once and repeatedly by two attending cardiologists who were blinded to the ECG findings of the patients, using a vivid S5 ultrasound machine (GE medical system, USA) with harmonic imaging. Left ventricular systolic function, defined by the left ventricular ejection fraction (LVEF), was calculated by the biplane Simpson’s method of discs. Coronary angiography was performed on an as-needed case-by-case basis by a team of expert cardiologists, in accordance with Judkins or Amplatz techniques. Multivessel disease was defined as the presence of luminal diameter stenosis of more than 50% in at least two major coronary arteries. Global Registry of Acute Coronary Events (GRACE) score was calculated for all patients [15].

### Statistical analysis

The normal distribution of all variables was tested by the Kolmogorov–Smirnov test. The median and interquartile range (IQR) of quantitative variables (due to non-normal distribution) and frequency and percentage of qualitative variables were reported. Kruskal–Wallis test was utilized to compare quantitative variables between groups. The Chi-square test was used for qualitative variables. Relative risk (RR) at 95% CI was calculated for each T wave amplitude strata. Sensitivity, specificity, negative and positive predictive values (NPV, PPV) were calculated for three cutoff points of T wave amplitude (0, − 1, − 2 mV), using MedCalc software version 19 (MedCalc Software Ltd, Belgium). Multivariable regression analysis was conducted to identify the independent determinants of T wave amplitude in aVR lead. In this analysis, T wave amplitude was considered as a continuous variable and the independent variables were selected from the most clinically relevant variables. All variables in a block were entered in a single step in the regression model (procedure for variable selection = Enter method). Before performing the modeling, continuous variables (including age, LVEF, Cr, BS, Systolic BP, HR) were converted to binary variables at their clinically relevant cut-off points (e.g., patients’ age was converted to the number of patients with the age of ≥ 65 years or < 65 years). Correlation between GRACE score and T wave amplitude on aVR lead was analyzed using Spearman's rank-order correlation test. Youden's J statistic (Youden’s index = sensitivity (%) + specificity (%) − 100) was used to determine the optimal cutoff point. The statistical performance of the GRACE score for prognosis of STEMI was calculated to be compared with that in T wave amplitude and better elucidate the prognostic value of T wave amplitude on aVR lead. For this purpose, receiver operating characteristic (ROC) curves are plotted with the calculation of concordance statistic (C-statistics = area under curve [AUC]) and coordinate points of ROC curve. The statistical analysis was conducted using SPSS version 24 (SPSS co., Chicago). Meta-analysis software (CMA) version 3 was used to subgroup analysis and forest plot. Two-sided P values (*p*) were reported with a significance level of 0.05. For the clinical outcome and relative risks, the statistical comparisons were conducted first between these four strata (≥ 0, − 1 to 0, − 2 to − 1, and < − 2 mV) with a focus on the group of T wave amplitudes of ≥ 0 mV as the most positive T wave group (Tables 1, 2). Then, three nominal cut-off points of T wave amplitudes (0 mV, − 1 mV, and − 2 mV) were selected and the discrimination power for each was determined (Table 3). Because of the highest Youden’s index at the cutoff point of − 1, subgroup analyses were conducted only at this level.

**Table 1:** Baseline characteristics and clinical outcomes of included STEMI patients

	Total n = 340	T < − n = 33	− 2 ≤ T < -− 1 n = 125	− 1 ≤ T < 0 n = 153	T ≥ 0 n = 29	*P* value
Age (years)	60 (51,70)	55 (48,59)	56 (48,67)	64 (54,72)	70 (63,78)	**0.001**
Gender n (%)						
Male	282 (82.9)	31 (93.9)	106 (84.8)	120 (78.4)	25 (86.2)	0.136
Female	58 (17.1)	2 (6.1)	19 (15.2)	33 (21.5)	4 (13.8)	
Diabetes n (%)	84 (24.7)	4 (12.1)	23 (18.4)	47 (30.7)	10 (34.4)	**0.019**
HTN n (%)	125 (36.7)	6 (18.1)	41 (32.8)	63 (41.1)	15 (51.7)	**0.020**
Positive FH n (%)	19 (5.5)	4 (12.1)	10 (8)	5 (3.2)	0 (0)	0.062
Previous PCI n (%)	9 (2.6)	0 (0)	4 (3.2)	5 (3.2)	0 (0)	0.559
Previous CABG n (%)	1 (0.2)	0 (0)	0 (0)	1 (0.6)	0 (0)	0.747
HC n (%)	56 (16.4)	4 (12.1)	18 (14.4)	30 (19.6)	4 (13.7)	0.554
HTG n (%)	30 (8.8)	2 (6)	18 (14.4)	7 (4.5)	3 (10.3)	**0.034**
Low HDL n (%)	51 (15)	6 (18.1)	21 (16.8)	23 (15)	1 (3.4)	0.306
Cr (mg/dl)	1.1 (0.9, 1.3)	1 (0.9, 1.1)	1.1 (1, 1.2)	1 (0.9, 1.3)	1.3 (1, 1.5)	**0.007**
Blood sugar (mg/dl)	140 (116.5, 181.5)	133 (101, 162)	141 (120, 177)	140 (115, 185)	168 (122, 192)	0.095
CKMB	166 (105, 271)	134 (87, 192)	171 (105, 271)	167 (107, 281)	185.5 (137.5, 403)	0.076
CTNI	16 (7,29)	11 (7,24)	15 (7,24)	15 (7,30)	24 (10,33.5)	**0.040**
Time from symptom to hospital (h)	3 (2, 7)	2 (2, 4)	3 (2, 4)	3 (2, 12)	6 (3, 14)	**0.001**
Systolic BP mmHg	115 (100, 140)	100 (100, 120)	120 (110, 140)	120 (100, 145)	110 (100, 140)	**0.016**
Diastolic BP mmHg	75 (70, 85)	70 (65, 75)	80 (70, 85)	75 (70, 85)	70 (65, 80)	**0.010**
HR/min	86 (75, 92)	74 (63, 85)	85 (74, 90)	90 (80, 95)	90 (85, 95)	**0.001**
Killip class n (%)						
1	211 (62.1)	25 (75.8)	94 (75.2)	85 (55.6)	7 (24.1)	**0.000**
> 1	129 (37.9)	8 (24.2)	31 (24.8)	68 (44.4)	22 (75.9)	
Infarction location n (%)						
ANT.SEPTAL	96 (28.2)	5 (15.1)	37 (29.6)	47 (30.7)	7 (24.1)	**0.000**
ANT	39 (11.4)	2 (6)	11 (8.8)	21 (13.7)	5 (17.2)	
EXT.ANT.LAT	68 (20)	2 (6)	21 (16.8)	34 (22.2)	11 (37.9)	
INF	84 (24.7)	20 (60.6)	36 (28.8)	25 (16.3)	3 (10.3)	
INF.POST	15 (4.4)	1 (3)	6 (4.8)	6 (3.9)	2 (6.8)	
INF.POST.LAT	16 (4.7)	1 (3)	10 (8)	5 (3.2)	0 (0)	
INF.RV	18 (5.2)	2 (6)	3 (2.4)	12 (7.8)	1 (3.4)	
INF.POST.RV	4 (1.1)	0 (0)	1 (0.8)	3 (1.9)	0 (0)	
LAT	0 (0)	0 (0)	0 (0)	0 (0)	0 (0)	
LVEF (%)	40 (35, 45)	45 (40, 50)	40 (35, 50)	40 (30, 45)	30 (25, 40)	**0.001**
MR n (%)						
No	125 (36.7)	15 (45.4)	51 (40.8)	53 (34.6)	6 (20.6)	**0.001**
Mild	184 (54.1)	18 (54.5)	69 (55.2)	82 (53.5)	15 (51.7)	
Moderate	31 (9.1)	0 (0)	5 (4)	18 (11.7)	8 (27.5)	
PAH n (%)						
No	305 (89.7)	33 (100)	119 (95.2)	131 (85.6)	22 (75.8)	**0.022**
Mild	32 (9.4)	0 (0)	6 (4.8)	19 (12.4)	7 (24.1)	
Moderate	2 (0.5)	0 (0)	0 (0)	2 (1.3)	0 (0)	
Severe	1 (0.2)	0 (0)	0 (0)	1 (0.6)	0 (0)	
Reperfusion strategy n (%)						
Fibrinolysis	171 (50.3)	15 (45.4)	75 (60)	68 (44.4)	13 (44.8)	0.101
PPCI	127 (37.3)	15 (45.4)	39 (31.2)	62 (40.5)	11 (37.9)	
Conservative treatment (no PPCI and no Fibrinolysis)^α^	42 (12.4)	3 (9.2)	11 (8.8)	23 (15.1)	5 (17.3)	
CAG results n (%)						
SVD	165 (48.5)	22 (66.7)	70 (56)	64 (41.8)	9 (31.0)	**0.021**
MultiVD	131 (38.5)	9 (27.2)	38 (30.4)	70 (45.8)	14 (48.3)	
Normal	8 (2.5)	0 (0.0)	5 (4.0)	3 (1.9)	0 (0)	
No CAG	36 (10.5)	2 (6.0)	12 (9.6)	16 (10.5)	6 (20.7)	
In-hospital mortality n (%)	24 (7)	0 (0)	1 (0.8)	16 (10.4)	7 (24.1)	**0.001**
Length of hospital stay (days)	6 (5, 7)	5 (5, 6)	6 (5, 7)	6 (5, 7)	7.5 (7, 10)	**0.001**
VT/VF n (%)	62 (18.2)	4 (12.1)	23 (18.4)	29 (18.9)	6 (20.6)	0.801
Re-hospitalization in 6 months^a^ n (%)	70 (22.2)	2 (6.1)	21 (16.9)	37 (27.0)	10 (45.5)	**0.001**
Six months cardiovascular mortality n (%)	52 (15.2)	1 (3.0)	6 (4.8)	32 (20.9)	13 (44.8)	**0.002**

The p-values lower than the significance level (0.05) were presented in bold

ANT, anterior; BP, blood pressure; CABG, coronary artery bypass grafting; CKMB, creatine kinase myocardial band; CTNI, cardiac troponin I; EXT.ANT.LAT, extensive anterolateral; FH, family history of ischemic heart disease; HC, hypercholesterolemia; HDL, high-density lipoprotein; HTG, hypertriglyceridemia; HR, heart rate; HTN, hypertension; INF, inferior; LAT, lateral; LVEF, left ventricular ejection fraction; MR, mitral regurgitation; PAH, pulmonary arterial hypertension; PCI, percutaneous coronary intervention; PPCI, primary percutaneous coronary intervention; POST, posterior; RV, right ventricular; Cr, creatinine; SVD, single-vessel disease; MultiVD, multi-vessel disease; CAG, coronary angiography

^a^The percentages were calculated out of 316 patients who were survived and successfully followed. α Due to contraindications fibrinolysis or PPCI were not perfomed and only medical treatment was administered

**Table 2:** Association of T wave amplitude in aVR lead for prognosis of in-hospital mortality, six month-cardiovascular mortality, and re-hospitalization in STEMI patients

T wave amplitude	In-hospital mortality			Six month-cardiovascular mortality			Re-hospitalization		
	RR	95% CI	ARF (%)	RR	95% CI	ARF (%)	RR	95% CI	ARF (%)
T ≥ 0	2.30	1.04–5.10	56.5	2.14	1.28–3.56	53.2	2.01	1.26–3.23	50.2
− 1 ≤ T < 0	1.00	Ref	Ref	1.00	Ref	Ref	1.00	Ref	Ref
− 2 ≤ T < -1	0.07	0.01–0.56	93	0.22	0.09–0.53	78	0.62	0.38–1.01	38
T < -2	< 0.01	–	> 99	0.14	0.02–1.02	86	0.22	0.05–0.88	78

ARF, absolute risk factor; Ref, utilized as reference group for calculation of RR and ARF

**Table 3:** Statistical measures of the performance of T wave amplitude in aVR lead at different cutoff points

	Six month-cardiovascular mortality		In-hospital mortality		Re-hospitalization	
	Value (%)	95% CI	Value (%)	95% CI	Value (%)	95% CI
Cutoff point of 0 mV						
Sensitivity	25.00	14.03–38.95	29.17	12.62–51.09	14.29	7.07–24.71
Specificity	94.44	91.13–96.79	93.04	89.65–95.59	95.12	91.63–97.45
PPV	44.83	29.37–61.35	24.14	13.15–40.07	45.45	27.32–64.88
NPV	87.46	85.60–89.11	94.53	93.03–95.73	79.59	77.92–81.16
Youden’s index	19.44		22.21		9.41	
Cutoff point of -1 mV						
Sensitivity	86.54	74.21–94.41	95.83	78.88–99.89	67.14	54.88–77.91
Specificity	52.43	46.49–58.32	49.68	44.04–55.33	54.47	48.02–60.81
PPV	24.73	21.84–27.86	12.64	11.19–14.24	29.56	25.32–34.19
NPV	95.57	91.48–97.75	99.37	95.83–99.91	85.35	80.35–89.25
Youden’s index	38.97		45.51		21.61	
Cutoff point of -2 mV						
Sensitivity	98.08	89.74–99.95	100.00	85.75–100.00	97.14	90.06–99.65
Specificity	11.11	7.73–15.32	10.44	7.30–14.35	12.60	8.73–17.41
PPV	16.61	15.85–17.40	7.82	7.55–8.09	24.03	22.91–25.18
NPV	96.97	81.72–99.57	100.0	–	93.94	79.18–98.44
Youden’s index	9.19		10.44		9.74	

PPV, positive predictive value; NPV, negative predictive value

## Results

### Baseline characteristics

The baseline clinical and laboratory data of 340 included STEMI patients and four T-wave strata are described in Table 1. T wave amplitudes of ≥ 0, − 1 to 0, − 2 to − 1, and < − 2 mV were detected in 29 (8.5%), 153 (45%), 125 (36.8%), and 33 (9.7%) patients, respectively. Age was significantly different among groups (*p*, 0.001): patients with a T wave amplitude of ≥ 0 mV had the highest median of age (median [IQR], 70 [63, 78] years in patients with T wave amplitude of ≥ 0 mV vs 64 [54, 72], 56 [48, 67], and 55 [48, 59] years in those with T wave amplitudes of − 1 to 0, − 2 to − 1, and < − 2 mV, respectively). The majority of patients were male (82.9%). There were no significant gender-specific differences between four T wave strata (*p*, 0.136). The prevalence of diabetes and hypertension (HTN) was significantly different among groups (*p*, 0.019 and 0.020, respectively) and they were more prevalent in patients with a T wave amplitude of ≥ 0 mV (34.5% and 51.7% in this group, respectively). Moreover, LVEF was significantly lower in patients with a T wave amplitude of ≥ 0 mV (median [IQR], 30 [25, 40]% in patients with T wave amplitude of ≥ 0 mV vs 40 [30, 45]%, 40 [35, 50]%, and 45 [40, 50] % in those with T wave amplitudes of − 1 to 0, − 2 to − 1, and < − 2 mV, respectively; *p*, 0.001).

### Clinical outcomes

An overall, 62 patients (18.2%) developed VT/VF, and 24 patients (7%) died during the hospital stay. In-hospital mortality was higher in patients with a T wave amplitude of ≥ 0 mV compared to other T-wave groups (7 [24.1%] in patients with T wave amplitude of ≥ 0 mV vs 16 [10.4%], 1 [0.8%], and 0 [0%] in those with T wave amplitudes of − 1 to 0, − 2 to − 1, and < − 2 mV, respectively; *p* 0.001, Table 1). However, no significant difference was observed between groups in terms of VT/VF rate (*p* 0.801). Length of hospital stay was also significantly different among groups (*p* 0.001). Patients with a T wave amplitude of ≥ 0 mV had significantly longer stay in hospital compared to other T-wave groups (median [IQR], 7.5 [7, 10] days in patients with a T wave amplitude of ≥ 0 mV vs. 6 [5, 7], 6 [5, 7], and 5 [5, 6] days in those with T wave amplitudes of − 1 to 0, − 2 to − 1, and < − 2 mV, respectively).

All the survived patients (316 cases) were successfully followed for six months. During this period, 70 patients (22.2%) were re-admitted to the hospital and 28 patients (8.8% of followed patients) died due to cardiovascular causes (total six months cardiovascular mortality, 52 cases [15.2%]). Both re-hospitalization and mortality were higher in those with a T wave amplitude of ≥ 0 mV (Table 1).

### Relative risks for clinical outcomes

**Fig. 1:**
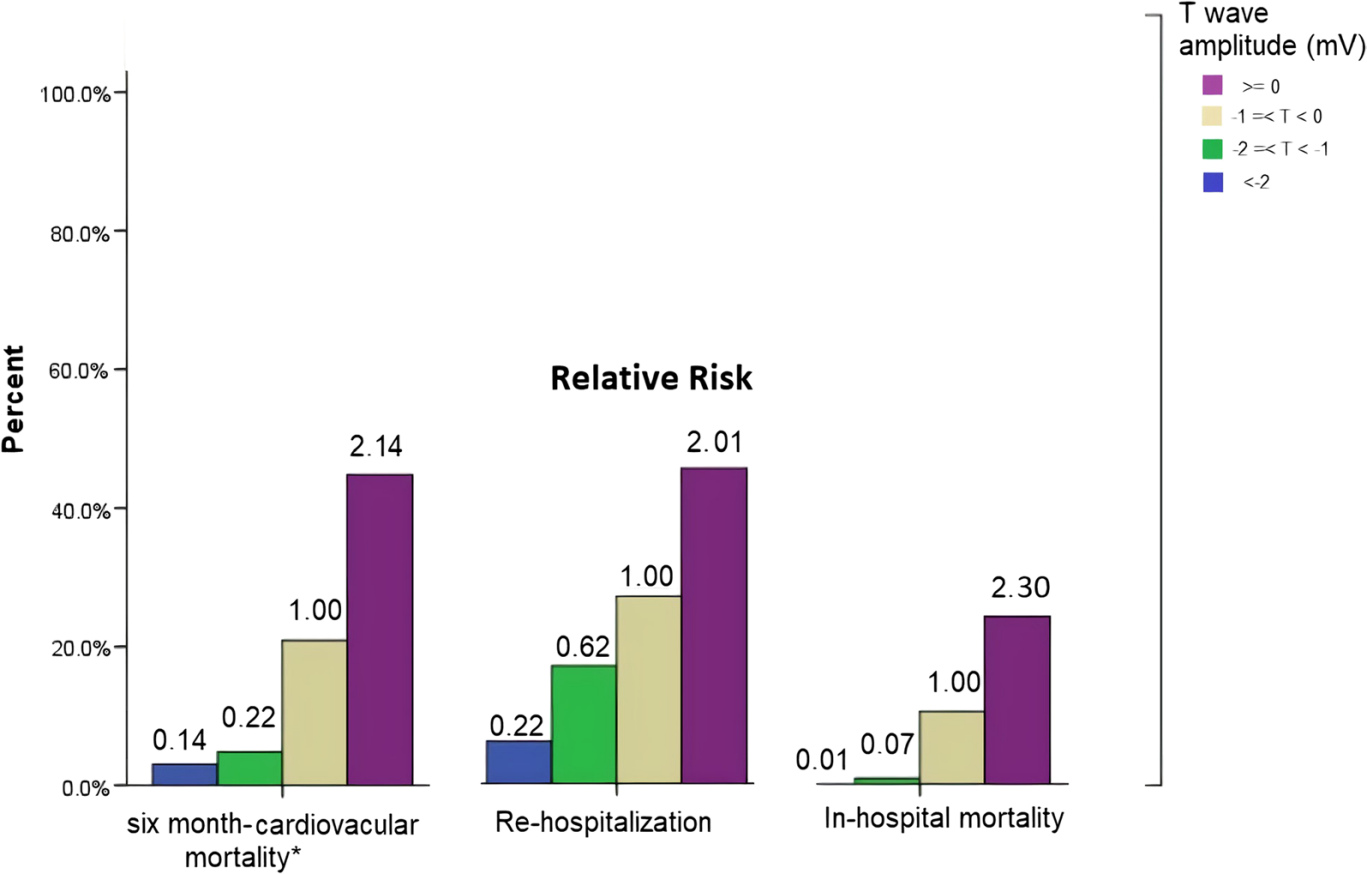
The rates of In-hospital mortality, Re-hospitalization, six month-cardiovascular mortality (including in-hospital deaths and six months after onset of disease) in different T wave amplitudes in aVR lead. Relative risks ratio values are shown above each bar. T wave amplitude of 0 to -1 mV was considered as the reference group

The relative risks (RR) for aVR lead T wave amplitude thresholds were calculated using the T-wave amplitude in aVR lead 0 to − 1 mV as the reference group since nearly half the population was in this group. The groups of patients with higher T wave amplitude in aVR, had progressively increased relative risk (RR) of in-hospital mortality, re-hospitalization, and six months-cardiovascular mortality (Fig. 1; Table 2). Moreover, there was a significant correlation between GRACE score and T wave amplitude on aVR lead (correlation coefficient [*β*], 0.354, *P* < 0.001, Additional file 1: Figure S3).

### Determinants of T wave amplitude in aVR lead

**Fig. 2:**
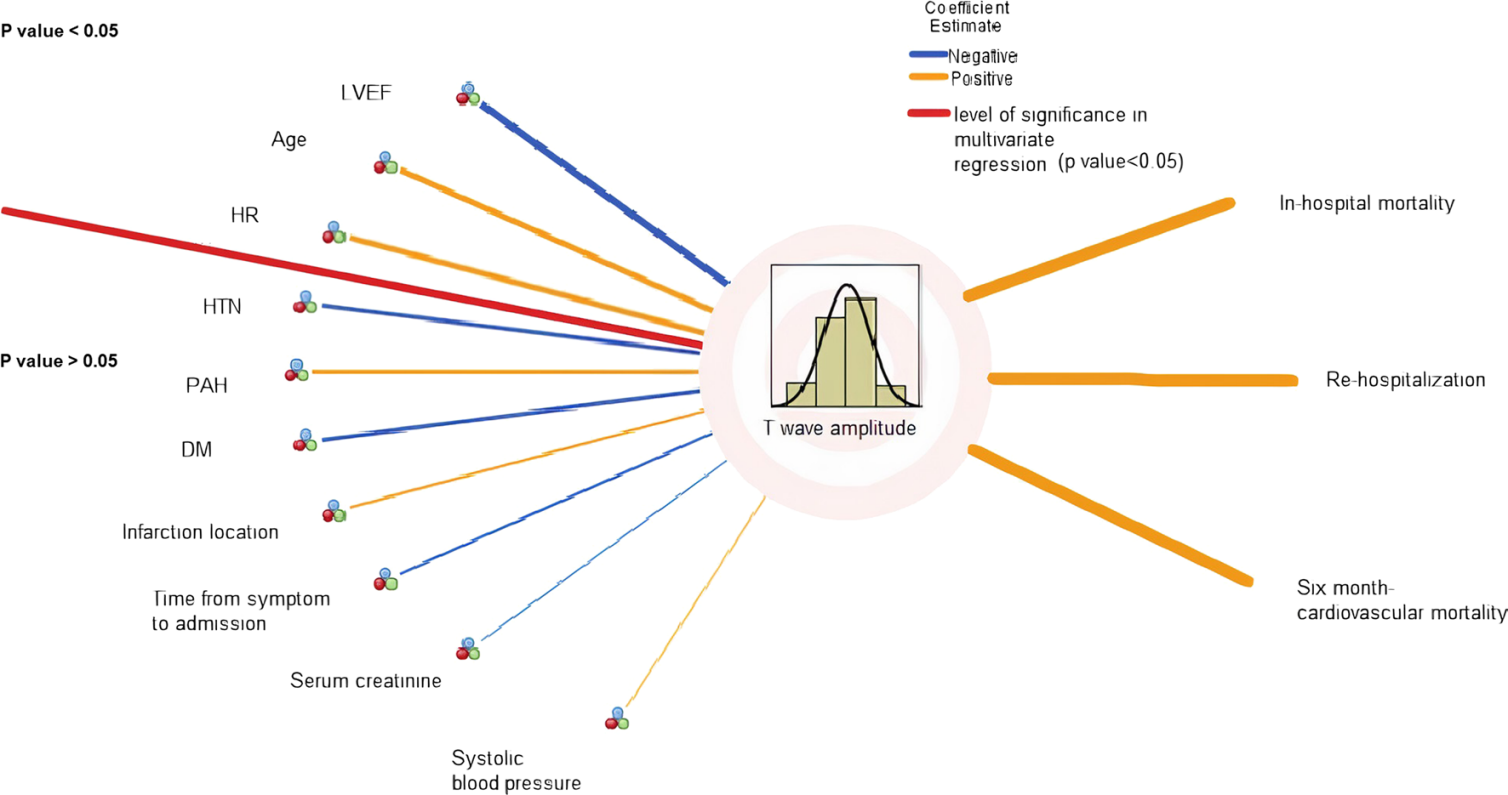
Summary of associations between T wave amplitude determinants and prognostic levels of T wave amplitude

The multivariate regression analysis identified three independent predictors of T wave amplitude in aVR lead including LVEF > 40% (*β* − 0.278; 95% CI − 0.477 to − 0.079), age ≥ 60 years (*β* 0.195; 95% CI 0.025–0.365), HR ≥ 90 (*β* 0.184; 95% CI 0.006–0.362). Other variables with non-significant correlations with T wave amplitude are described in Additional file 1: Table S1. Associations between T wave amplitude in aVR lead determinants and their correlations with patients' clinical outcomes are depicted in Fig. 2.

### Performance of T wave amplitude in aVR lead for prediction of the short and midterm clinical outcomes versus GRACE score

T wave amplitude at the cutoff point of 0 mV had high specificity (> 90%), but low sensitivity (< 50%) for prediction of the endpoints (including 6-month cardiovascular mortality, in-hospital mortality, and re-hospitalization (Additional file 1: Table S2 and Table S3). On the other hand, T wave amplitude at the cutoff points of − 1 mV and − 2 mV exhibited high sensitivity (> 90%), but low specificity (< 60%) for the prediction of all study endpoints (Table 3). The cutoff point of − 1 mV had the highest Youden’s index value for all study endpoints. The sensitivity and specificity of T wave amplitude at the cutoff point of -1 mV were 86.54% (95% CI 74.21–94.41) and 52.43% (95% CI 46.49–58.32) for cardiovascular mortality in six months, 95.83% (95% CI 78.88–99.89) and 49.68% (95% CI 44.04–55.33) for in-hospital mortality, and 67.14% (95% CI 54.88–77.91) and 54.47% (48.02–60.81) for re-hospitalization, respectively (Table 3).

Nevertheless, subgroup analysis demonstrated that T wave amplitude at the cutoff point of − 1 mV had higher specificity in those patients with better clinical conditions (age < 65 years, non-diabetic, non-hypertensive, with Cr < 1.2 mg/dl, early presentation [time from symptom to hospital < 12 h, Killip class = 1, or having no PAH or MR, Table 4).

**Table 4:** Subgroup analysis for statistical measures of the performance of T wave amplitude in aVR lead at the cutoff point of -1 mV for prediction of in-hospital mortality

	Number of in-hospital mortality/total		Sensitivity	Specificity	Relative risk and 95%CI
	T wave ≥ -1 mV	T wave < -1 mV			
All patients	23/182	1/158	95.83	49.68	19.96 (2.72, 146.17)
Age (years)					
< 65	6/89	1/113	85.71	57.44	7.61 (0.93, 62.12)
≥ 65	17/93	0/45	100.00	37.19	17.12 (1.05, 278.55)
Gender					
Female	6/37	0/21	100.00	40.38	7.52 (0.44, 127.29)
Male	17/145	1/137	94.44	51.52	16.06 (2.16, 119.06)
DM					
Absent	16/125	1/131	94.12	54.39	16.76 (2.25, 124.56)
Present	7/57	0/27	100.00	35.06	7.24 (0.42, 122.33)
HTN					
Absent	11/104	1/111	91.67	54.19	11.74 (1.54, 89.35)
Present	12/78	0/47	100.00	41.59	15.18 (0.92, 250.75)
HC					
Absent	19/148	1/136	95.00	51.14	17.45 (2.36, 128.66)
Present	4/34	0/22	100.00	42.31	5.91 (0.33, 104.72)
Cr (mg/dl)					
< 1.2	6/109	0/109	100.00	51.42	13.00 (0.74, 227.97)
≥ 1.2	17/73	1/49	94.44	46.15	11.41 (1.56, 82.98)
BS (mg/dl)					
< 200	14/143	0/133	100.00	50.76	26.98 (1.62, 447.95)
≥ 200	9/39	1/25	90.00	44.44	5.76 (0.77, 42.8)
Time from symptom^a^ (h)					
< 12	14/130	0/131	100.00	53.04	29.22 (1.76, 484.78)
≥ 12	9/52	1/27	90.00	37.68	4.67 (0.62, 34.98)
Systolic BP mmHg					
< 100	18/23	1/9	94.74	61.54	7.04 (1.09, 45.26)
≥ 100	5/159	0/149	100.00	49.17	10.31 (0.57, 184.9)
HR/ min					
< 90	16/96	1/47	94.12	36.51	7.83 (1.07, 57.3)
≥ 90	7/86	0/111	100.00	58.42	19.31 (1.11, 333.49)
Killip class					
1	1/93	0/119	100.00	56.40	3.83 (0.15, 92.94)
> 1	22/89	1/39	95.65	36.19	9.64 (1.34, 69.01)
Infarction location					
Anterior	17/125	1/78	94.44	41.62	10.60 (1.44, 78.13)
Inferior	6/57	0/80	100.00	61.07	18.15 (1.04, 315.94)
PAH					
Absent	15/153	1/152	93.75	52.25	14.90 (1.99, 111.41)
Present	8/29	0/6	100.00	22.22	3.96 (0.25, 60.87)
MR mild to moderate	20/123	1/92	95.24	46.91	14.95 (2.04, 109.44)
No MR	3/59	0/66	100.00	54.10	7.81 (0.41, 148.24)
LVEF (%)					
< 40	23/96	1/38	95.83	33.64	9.10 (1.27, 65.05)
≥ 40	0/86	0 /120	–	58.25	–

BP, blood pressure; CABG, coronary artery bypass grafting; FH, family history of ischemic heart disease; CKMB, creatine kinase myocardial band; CTNI, cardiac troponin I; HC, hypercholesterolemia; HTN, hypertension; HTG, hypertriglyceridemia; HDL, high-density lipoprotein; HR, heart rate; LVEF, left ventricular ejection fraction; MR, mitral regurgitation; PAH, pulmonary arterial; PCI, percutaneous coronary intervention; Cr, creatinine

^a^Time from symptom to hospital admission

GRACE score indicated higher discriminative potential of prognosis toward the study endpoints (Additional file 1: Figure S4). It had an AUC of 0.928 (95% CI 0.883–0.973) for in-hospital mortality (optimal cutoff point, 167 with sensitivity and specificity of 83.3% and 83.7%, respectively), 0.935 (95% CI 0.906–0.963) for six month-cardiovascular mortality (optimal cutoff point, 141 with sensitivity and specificity of 90.4% and 93.0%, respectively), and 0.757 (95% CI 0.688–0.826) for re-hospitalization (optimal cutoff point, 149 with sensitivity and specificity of 50.0% and 93.3%, respectively).

## Discussion

Adverse clinical outcomes including mortality of inpatients, length of hospitalization, re-hospitalization, and cardiovascular mortality in six months were higher (> twofold for all these endpoints) for people with a T wave amplitude of ≥ 0 mV compared to other T-wave groups (T wave amplitude − 1 to 0, − 2 to − 1, and < − 2 mV). The groups of patients with higher T wave amplitude in aVR (from T wave amplitude of under − 2 mV to − 2 to − 1 mV, and subsequently to − 1 to 0 mV, and higher than 0 mV), had progressively increased risk of in-hospital mortality, re-hospitalization, and six month-cardiovascular mortality. However, no significant differences were observed between groups in terms of VT/VF rate. Consistent with our findings, Ayhan et al. demonstrated that positive T wave in lead aVR on admission ECG (≥ 0.1 mV) was associated with in-hospital mortality in 169 patients with anterior wall STEMI treated with primary percutaneous coronary intervention (OR 4.41, 95% CI 1.2–22.1; *p* 0.05), although in this study patients were not followed up [16]. Others pointed out that the presence of a positive T wave in lead aVR is associated with an unstable clinical condition [12, 17]. Shinozaki et al. postulated that T wave in lead aVR was related to higher pulmonary arterial, pulmonary capillary wedge, and left ventricular end-diastolic pressures and a severely reduced cardiac function in patients with anterior wall old myocardial infarction [12]. Furthermore, the association of positive T wave in lead aVR with cardiovascular mortality is not limited to those presumably with a myocardial infarction. Anttila et al., by evaluation of standard ECGs obtained from the general population (6354 people) from a large nationally representative health examination survey, reported that positive T wave in lead aVR (≥ 0 mV) was significantly correlated with both cardiac and all-cause mortality during the median follow-up of 98.5 months [17]. Likewise, in a retrospective study of 24,270 male veterans’ ECGs which were obtained for different clinical reasons, Tan et al. reported that positive T wave in lead aVR (≥ 0 mV) was related to the increased cardiovascular mortality in six months (24% vs 7.7% for entire study population) and a fivefold increased relative risk of mortality during 7.5-year follow-up [5]. Similar observations were also reported for people with a positive T wave in lead aVR (> − 0.01 mV) in 7928 participants enrolled in the National Health and Nutrition Examination Survey III, California (13.5 ± 3.8 years of follow-up) [18].

In our study, there was a significant difference between four T wave strata in terms of coronary angiography results. Of note, multivessel involvement was more prevalent in the groups with a T wave amplitude of -1 to 0 mV and ≥ 0 mV than those with T wave amplitude less than − 1. Consistent with this, Ayhan et al. demonstrated that a positive T wave in lead aVR in patients with anterior STEMI is associated with multivessel disease (14). Despite that the exact mechanism of appearance of the positive T wave in lead aVR remains to be elucidated, some investigators have postulated that multivessel coronary artery disease, causing the injury to the apical, inferior, and lower lateral regions of the heart, bring about the deviation of the vector of the T wave towards the injured region and leads to a flat or positive T wave [12, 19]. The incidence of cardiogenic shock, hemodynamic collapse, and mortality are higher in patients with multivessel coronary artery disease who have developed STEMI [19]. Therefore, one of the explanations of poor prognosis in STEMI patients with positive T wave in aVR may be because of multivessel involvement.

Patients in different T wave strata in our study differed in several characteristics as follows: Those with T wave ≥ 0 mV were older adults with later presentation, a worse condition in presentation [higher Killip class], worse mitral regurgitation, and higher pulmonary arterial hypertension (Table 1). These variations were consistent with the reports of the previous studies [5, 17]. Möller et al. demonstrated that the prevalence of T-wave abnormalities in the general population rises by advancing age, being 5.9% at 50 years of age and 16% at 70 years of age [20]. In our study, those exhibiting several other conditions (such as diabetes, HTN, elevated levels of serum Cr and CTNI, low LVEF) were more prevalent among patients with T wave ≥ 0 mV. Therefore, we assumed that positive T wave in aVR lead is due to these worse conditions and performed a multivariate regression analysis to identify the independent determinants of developing positive T wave in aVR lead. The multivariable model demonstrated that certain conditions (e.g., older age, lower LVEF, higher HR) on admission were independently associated with the development of positive T wave in aVR lead. These conditions have been previously recognized as the most important clinical indicators of high risk patients in the acute phase of STEMI [13]. Therefore, patients with worse symptoms and high risk were more prone to show positive T wave in aVR lead. Another explanation is that positive T wave appears to be a repolarization defect that is more often present in patients with older age, larger infarcts size, later presentation, low LVEF, each or taken together can represent adverse outcome (Additional file 1: Table S1). This finding was further supported by the significantly positive correlation between T wave amplitude with GRACE score which has been previously validated and demonstrated good discriminative potential for 6-month mortality (C-statistic = 0.81) [15].

Conceivably, T wave change in aVR lead during either anterior wall MI or inferior MI could exhibit different critical characteristics, should it represent the territory at risk during STEMI. In our study, more patients with anterior wall MI had T wave amplitude of > 0 mV. In multivariate regression analysis, 'infarct location' appears not to be an independent determinant of the positive T wave in aVR lead (Additional file 1: Table S1). Furthermore, in subgroup analyses, the sensitivity and specificity of T wave for prediction of in-hospital mortality, as well as its relative risk in subgroups of anterior and inferior MI, were comparable (Table 4). Therefore, this repolarization deviation (positive T wave) does not accompany the infarct location.

Our study was the first that evaluated the discriminative potential of T wave amplitude in aVR lead for prognosis of STEMI. Despite the higher rate of major adverse events (in-hospital mortality, re-hospitalization, cardiovascular mortality in six months)—in patients with a T wave amplitude of ≥ 0 mV and T wave amplitude − 1 to 0 compared to other T-wave groups (− 2 to − 1, and < − 2 mV)—the prognostic value of T-wave amplitude at different cut-off points was limited by either low sensitivity or low specificity. Regarding the results of the Youden’s index, the cutoff point of − 1 mV was the optimal point; however, the prognostic value of T wave amplitude in this cutoff point was limited by low specificity (around 50%). Because sensitivity or specificity near the cutoff remained low, those who had poor prognosis could incorrectly be labeled as good prognosis or vice versa. The better test to use to rule out a poor prognosis is the one with a smaller likelihood ratio of a negative test. However, aVR measure appears crucial, particularly for cases detected with early signs of severe clinical conditions.

Notably, predetermined criteria in clinical investigations remain the gold standard and can provide a deeper understanding toward further medical counseling. Furthermore, repeat aVR tests might reduce an intrinsic ‘lead time bias’ and ‘length bias’ in prognosis and thus facilitate managing quality-of-life in progression-free or disease-free survival, when utilized in connection with appropriate therapy.

The GRACE score which is widely accepted as an accurate scale for risk assessment of STEMI patients exhibited a higher discriminative potential than the T wave in lead aVR toward the adverse clinical outcome. Therefore, the GRACE score appears as a useful auxiliary option toward a better clinical judgment for STEMI patients.

Although a broad consensus is currently unavailable, other ECG changes (e.g., deviations in P wave, PR interval, QRS interval, Q-wave) have been proposed for the diagnosis and management of patients with coronary artery disease [21, 22]. Of note, the ST-segment deviation (in aVR as well as in other ECG leads), with its high specificity and availability, is one of the essential ECG parameters for the diagnosis of the acute coronary syndrome and management of patients by localizing the culprit coronary artery and the site of occlusion (proximal versus distal), predicting outcomes, and evaluating treatment success/failure [21, 23, 24].

### Study limitations

Although our study was the first to investigate the short and midterm prognosis of STEMI patients with positive T wave in aVR and its discriminative potential, it contains certain limitations. The diagnosis of STEMI was made according to recent guidelines [13]. Neither the invasive procedures (such as tissue histopathology examination), nor provocative testing for ischemia or nuclear magnetic resonance imaging were conducted to validate the diagnosis and the affected myocardium (infarction location). To be more practical in interpretations of ECG records by the clinicians and to make it readily observable independently, especially in an emergency room situation, the smallest unit of T wave amplitude change was considered as 1 mV. T wave amplitude as continuous variables (for values smaller than 1 mV) is questionable relative to commonly used blood tests. Future investigations should verify the sufficiency, accuracy, and precision of the proposed optimal cutoff points for T wave amplitude for a better threshold prognosis, particularly for disconcordants, as well as people with unverified clinical characteristics and treatments in relation to multiple risk factors (Fig. 2; Additional file 1: Table S1). Prognosis of the uncertain anterior STEMI should be confirmed through exclusionary diagnosis of non-ST segment (NSTEMI) cases in high risk people that typically require interventions (e.g., blood thinners, PCI, stenting) with independent variables and those with blockages of multiple coronary arteries and diabetes that CABG rather than angioplasty is recommended.

## Conclusion

Our study demonstrated a significant association of positive T wave in aVR lead and risk of adverse clinical outcome including in-hospital mortality, length of stay, as well as six-month cardiovascular mortality and re-hospitalization in STEMI patients. Nevertheless, the prognostic value of T-wave amplitude at different cut-off points was limited by low discriminative potential (either low sensitivity or low specificity). Therefore, clinicians should be attentive to these markers and limitations upon the interpretation of aVR lead T-wave amplitude in STEMI patients and the clinical decision should not be based on merely the T-wave amplitude. The GRACE score serves as a useful add-on scale for risk assessment and determination of reasonable prognosis in STEMI patients.

## Supplementary Information

**Additional file 1.** The supplemental tables and figures.

## Data Availability

All Data and material collected during this study are available from the corresponding author upon reasonable request.
