# Supplementary material for: Clinical utility of aVR lead T-wave in electrocardiogram of patients with ST-elevation myocardial infarction
Source: BMC Cardiovasc Disord. 2021 Oct 28;21:520. doi: 10.1186/s12872-021-02335-5 (PMC8555143; doi:10.1186/s12872-021-02335-5)
Supplement: Supplementary file 1 — Additional file 1. The supplemental tables and figures. [file 12872_2021_2335_MOESM1_ESM.docx]

**Supplemental Material**

**Clinical Utility of aVR Lead T-wave in Electrocardiogram of Patients with ST-Elevation Myocardial Infarction**

[Authors' names/affiliations are undisclosed for peer review]

*Running title*: *T-wave in aVR Lead in ST-Elevation Myocardial Infarction*

|  | Supplemental Table 1. Independent determinants of T wave amplitude in aVR lead in STEMI patients | | | | |
| --- | --- | --- | --- | --- | --- |
|  | |  | **Correlation coefficient and 95%CI** | **P value** |  |
| Age | | **>= 65 years vs <65 years** | 0.195 (0.023, 0.357) | .**026** |  |
| Gender | | **Male vs female** | -0.012 (-0.224, 0.201) | .913 |  |
| DM | | **Present vs absent** | -0.186 (-0.394, 0.023) | .075 |  |
| HTN | | **Present vs absent** | -0.159 (-0.322, 0.004) | .072 |  |
| HC | | **Present vs absent** | -0.067 (-0.276, 0.141) | .526 |  |
| Cr (mg/dl) | | **>=1.2 vs <1.2** | -0.090 (-0.273, 0.093) | .321 |  |
| BS (mg/dl) | | **>=200 vs <200** | -0.037 (-0.267, 0.193) | .754 |  |
| Time from symptom ^a^ (hours) | | **>=12 vs <12** | -0.135 (-0.317, 0.048) | .136 |  |
| Systolic BP mmHg | | **>=100 vs 100** | 0.107 (-0.170, 0.384) | .448 |  |
| HR/ min | | **>=90 vs <90** | 0.184 (0.010, 0.364) | **.038** |  |
| Killip class | | **> 1 vs <1** | 0.073 (-0.121, 0.267) | .815 |  |
| Infarction location | | **Inferior vs anterior** | 0.144 (-0.044, 0.333) | .116 |  |
| PAH | | **Present vs absent** | 0.248 (-0.015, 0.512) | .057 |  |
| MR | | **No MR vs MR** | -0.042 (-0.209, 0.126) | .625 |  |
| LVEF (%) | | **>=40 vs <40** | -0.253 (-0.050, -0.456) | **.015** |  |
|  | |  |  |  |  |
| a Time from symptom to hospital admission; BP, Blood pressure; HC, Hypercholesterolemia; HTN, Hypertension; HR, Heart rate; LVEF, Left ventricular ejection fraction; MR, Mitral regurgitation; PAH, Pulmonary arterial;  PCI, Percutaneous coronary intervention | | | | |  |

**a** Numbers of patients in this category (percent in row)

**b** Of those discharged from the hospital (n=316)

| Supplemental Table 2 . Performance of T wave amplitude in aVR lead at different cutoff points for prognosis of distinct endpoints in STEMI patients | | | | | | | | | | |
| --- | --- | --- | --- | --- | --- | --- | --- | --- | --- | --- |
|  | **T** **wave**  **amplitude mV** | **Six month-cardiovascular mortality**  **n = 340** | | | **In-hospital mortality**  **n = 340** | | | **Re-hospitalization**  **n = 316^b^** | | |
|  |  | **Present** | **Absent** | **Present** | | **Absent** | **Present** | | **Absent** |  |
| Cutoff point of 0 mV | ≥0 | 13 (44.8)^a^ | 16 (55.2) | 7 (24.1) | | 22 (75.9) | 10 (45.5) | | 12 (54.5) |  |
|  | <0 | 39 (12.5) | 272 (87.5) | 17 (5.5) | | 294 (94.5) | 60 (20.4) | | 234 (79.6) |  |
| Cutoff point of -1 mV | -1≤ | 45 (24.7) | 137 (75.3) | 23 (12.6) | | 159 (87.4) | 47 (29.6) | | 112 (70.4) |  |
|  | <-1 | 7 (4.4) | 151 (95.6) | 1 (0.6) | | 157 (99.4) | 23 (14.6) | | 134 (85.4) |  |
| Cutoff point of -2 mV | -2≤ | 51 (16.6) | 256 (83.4) | 24 (7.8) | | 283 (92.2) | 68 (24.0) | | 215 (76.0) |  |
|  | <-2 | 1 (3.0) | 32 (97.0) | 0 (0.0) | | 33 (100.0) | 2 (6.1) | | 31 (93.9) |  |

**Supplemental Figure 1**. STARD diagram reporting flow of participants through the study


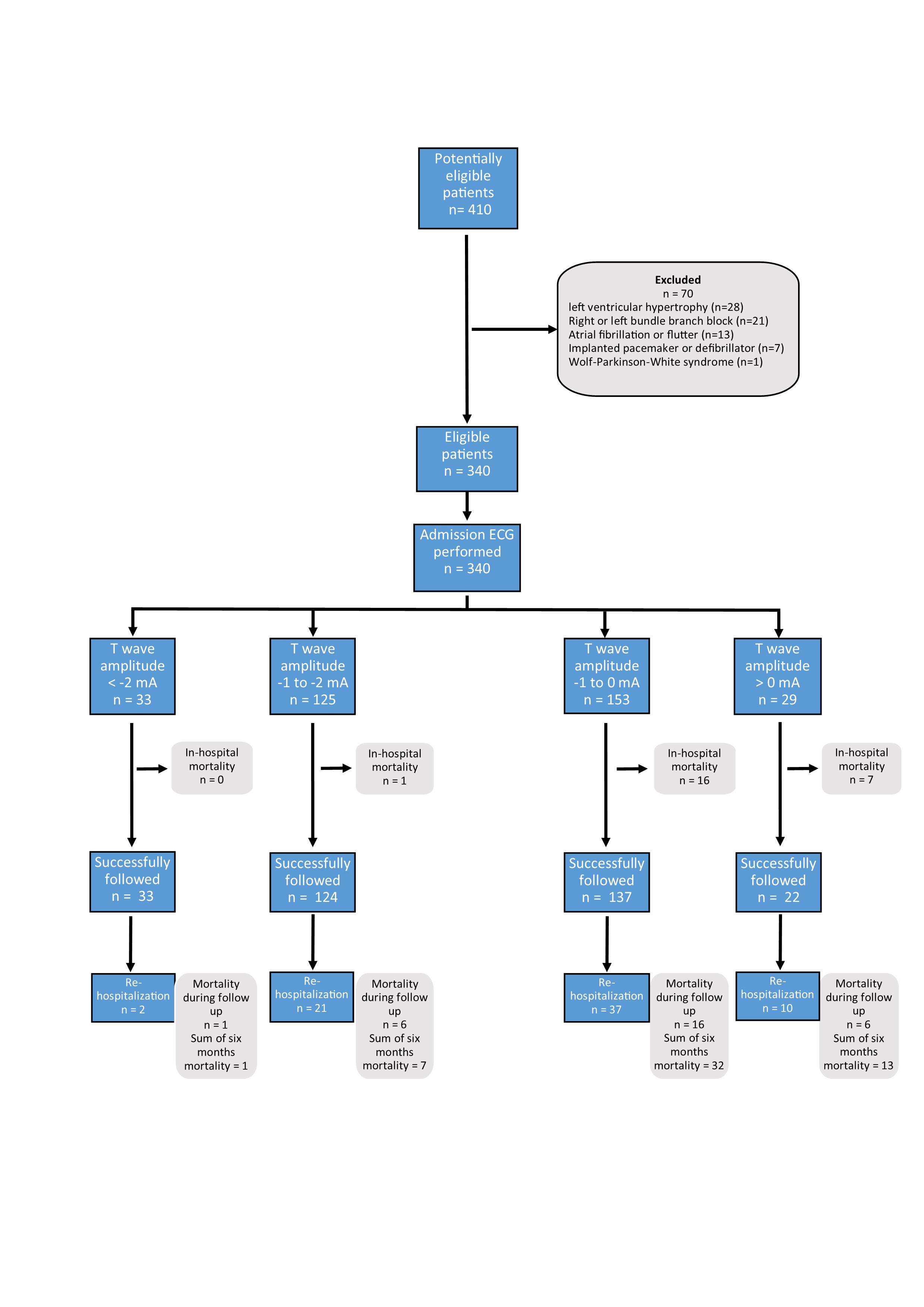


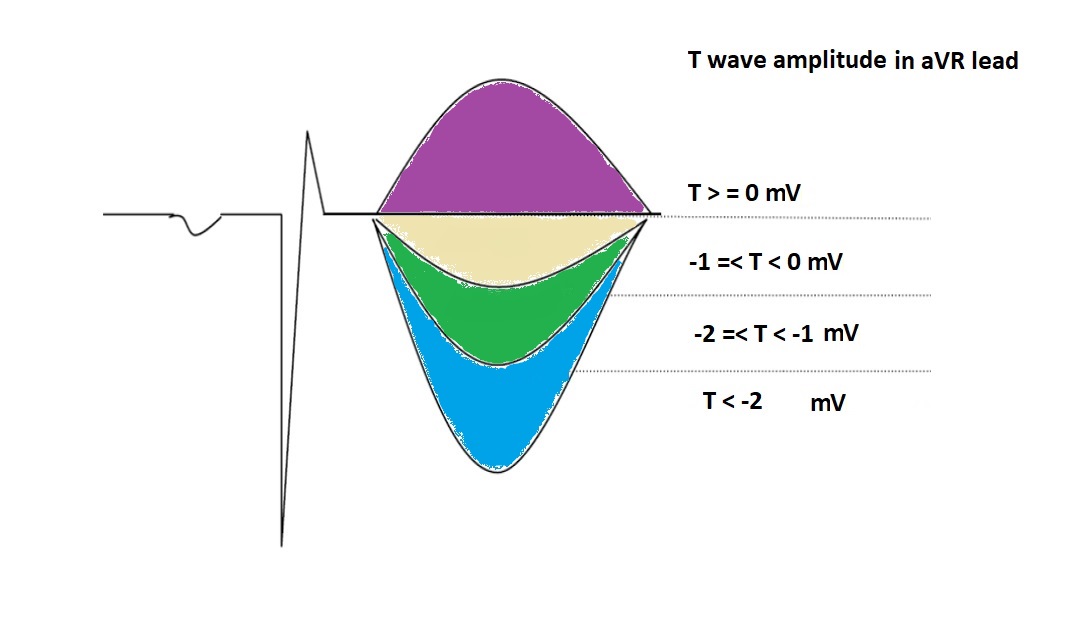


**Supplemental Figure 2**. Study groups based on T-wave amplitude.


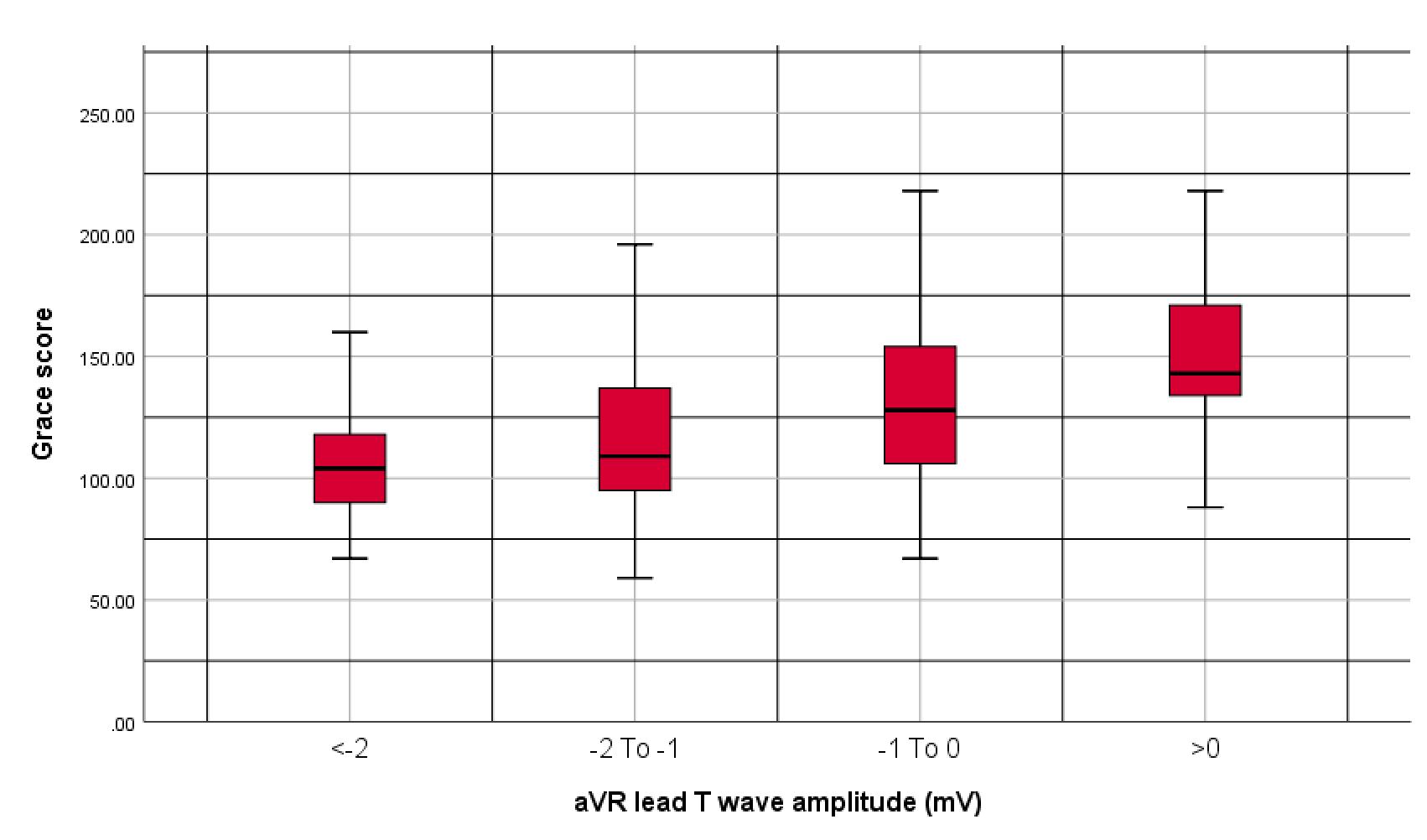
 **Supplemental Figure 3.** Simple boxplot of Global Registry of Acute Coronary Events (GRACE) score in patients with different T wave amplitude in aVR lead.


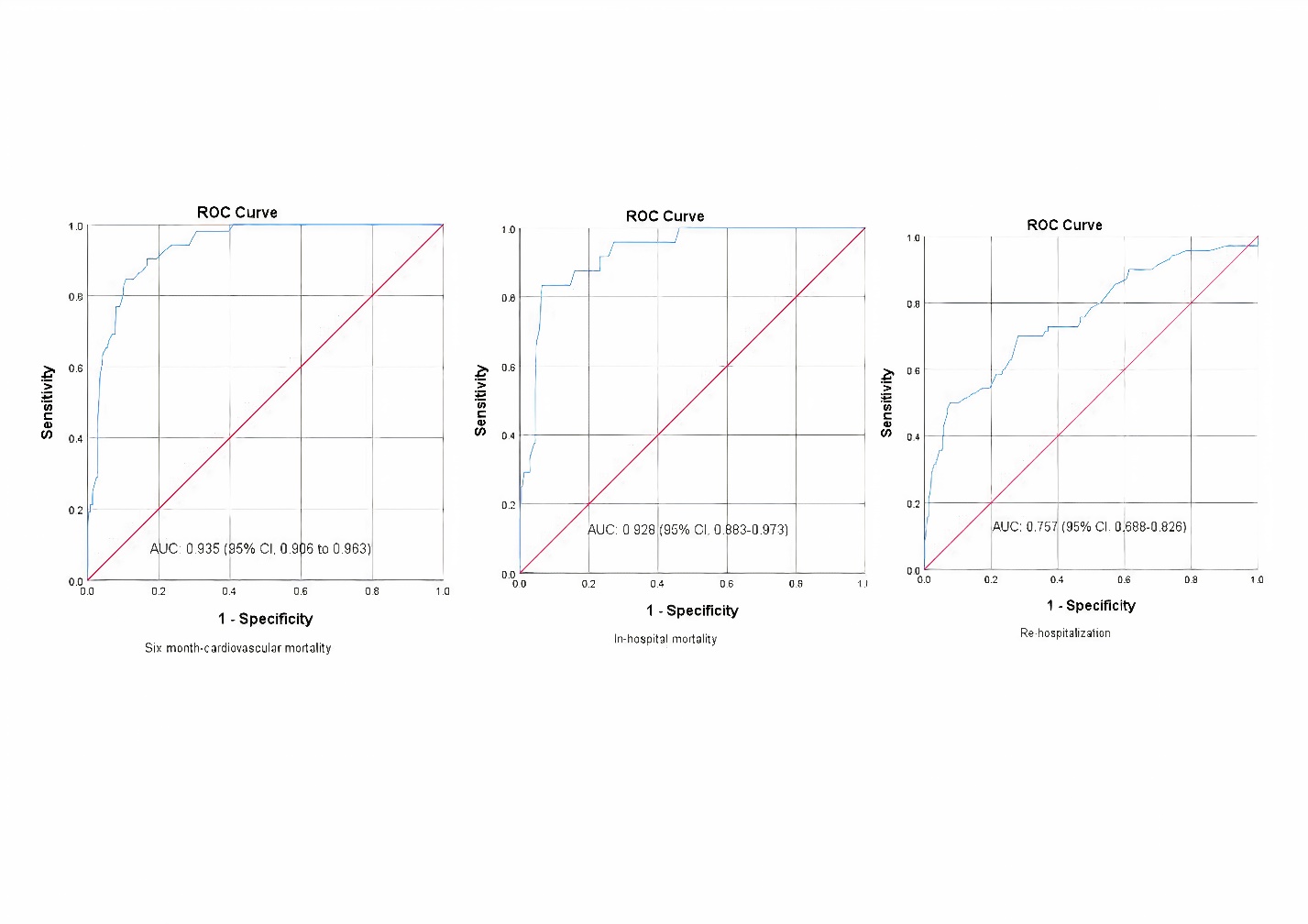


**Supplemental Figure 4**. Receiver Operating Characteristic (ROC) curve analysis for prognostic power of Global Registry of Acute Coronary Events (GRACE) score in patients with different T wave amplitude in aVR lead
